# *Metabolites*: A Novel Platform for Converging Research on Metabolism and Metabolomics

**DOI:** 10.3390/metabo1010001

**Published:** 2010-12-07

**Authors:** Vladimir A. Likić

**Affiliations:** Bio21 Molecular Science and Biotechnology Institute, University of Melbourne, 30 Flemington Road, Parkville, VIC 3010, Australia; E-Mail: vlikic@unimelb.edu.au

Technological advances in analytical instrumentation and advances in data modeling are working in synergy to open up new perspectives and research agendas in metabolic research. Thanks to the legacy of the Human Genome Project and its continued impact in the post-genomic era, metabolism is now thought of in a whole-genome context, even when the focus is on a single metabolite and individual metabolic reactions. For a few model organisms we now have extensive, and in some cases complete, information about components that perform integrated metabolic functions. This promises a true paradigm shift in our understanding of the processes of metabolism, but also poses new challenges. As complex and coordinated global behaviors are observed in what were thought to be “simple” organisms, many challenges remain in the experimental domain, as well as in the integration of data generated by increasingly high-throughput analytical techniques. Indeed, in the new era of metabolic research, mathematical and computational modeling is expected to play an increasingly important role. For many complex biochemical phenomena, use of mathematical models may be the best way to build a consistent picture and generate testable hypotheses based on complex yet inevitably incomplete data sets.

Development of new therapies for many human and animal diseases depends on a better understanding of metabolic pathways, their control and regulation. The awareness of the general public about the relationship between diet and major ailments of modern society, such as diabetes, obesity, cardiovascular diseases, and others, means that metabolism is truly in the public eye. The awareness of policy makers of these issues means that more funding is becoming available for novel, visionary, post-genomic era metabolic research. Most drugs and many clinical biomarkers are small molecules, and a number of simple metabolite biomarkers have been in use for decades to assess disease or the risk of disease. Development of new monitoring, preventive, and early detection measures for many health conditions largely depends on finding new small molecule biomarkers, and on understanding their biological roles and their correlated responses. And when it comes to biotechnology and metabolic engineering, development of more efficient, cleaner manufacturing practices in the production of many precious chemicals depends on a better understanding of metabolic processes in industrial microorganisms. The perspective of *de novo* design of biosynthetic pathways, *in silico* modeling of metabolism, and systems approaches to strain improvement are only some of the new, exciting frontiers.

*Metabolites* is a new journal from MDPI concerned with molecular and biological aspects of metabolism, relevant to the fields of metabolomics, metabolic biochemistry, biotechnology, and medicine, with a focus on metabolites and small molecule biomarkers. *Metabolites* aims to provide an international forum for exchange of ideas and results focused on small molecule components in biological systems, their function, distribution, and regulation. The launch of a new journal focusing on small molecules and their roles in the processes of metabolism is timely. In the past decade, we have witnessed steady advances in methods for the detection and identification of small molecules, with ever increasing sensitivity and comprehensiveness of detection. *Metabolites* aims to emphasize the science at the interface of analytical chemistry, biochemistry, and computer and information science, with the recognition that further development of both analytical and computational methods will be necessary for new breakthroughs in our understanding of biological roles of small molecules. *Metabolites* therefore aims to track the evolving technologies for small molecule detection and identification, computational methods for data analysis and integration, and the application of these methods in metabolic research. A prompt, yet rigorous peer review process, rapid dissemination practices, and the open access policy will ensure the widest possible audience for all papers published by *Metabolites*.

The topics of interest to *Metabolites* include identification and quantification of endogenous and exogenous metabolites in unicellular and multicellular organisms, including lipidomics; downstream effects of genetic and environmental perturbations on metabolite levels and fluxes; and advances in methods and platform technologies for low-molecular weight molecules detection and identification. *Metabolites* encourages contributions in computational and theoretical aspects relevant to the understanding of metabolic processes and metabolic networks, such as metabolic reconstructions and computational models of metabolic processes; advances in the capture and organization of metabolic knowledge; the analysis of metabolic flux; and advances in data processing relevant to detection and identification of low-molecular weight molecules. Finally, *Metabolites* will publish applications of these techniques and methodologies in microorganisms, including metabolic engineering and studies of microbial pathogens; in plant, environmental, and nutritional sciences; in animals for metabolism studies; and in the clinical laboratory where the aim is biomarker discovery and understanding of metabolic diseases.

